# Supplementary material for: Flattening the Curve after the Initial Outbreak of Coronavirus Disease 2019: A Data-Driven Modeling Analysis for the Omicron Pandemic in China
Source: Vaccines (Basel). 2023 May 22;11(5):1009. doi: 10.3390/vaccines11051009 (PMC10224169; doi:10.3390/vaccines11051009)
Supplement: Supplementary file 1 [file vaccines-11-01009-s001.zip › vaccines-2289768-supplementary.pdf]

Table S1. Vaccine efficacy assumption

	Outcome	Susceptible population	People got the first shot	First shot activated	People got the first shot and were ready for the second shot	People got the second shot	Second shot activated	People got the second shot and were ready for the booster (waning status)	People got booster vaccination	Booster shot activated	Waning status after booster vaccination	People recovered from the Omicron infection	People recovered from the Delta infection
Inactivated	Infection	100.0%	100.0%	94.4%	94.4%	94.4%	90.9%	94.1%	94.1%	83.0%	86.2%	9.5%	43.5%
	Onward transmission	100.0%	100.0%	100.0%	100.0%	100.0%	100.0%	100.0%	100.0%	89.4%	100.0%	100.0%	100.0%
	Symptomatic desease	100.0%	100.0%	88.5%	88.5%	88.5%	80.4%	87.9%	87.9%	64.5%	72.2%	3.4%	6.7%
	Hospitalization	100.0%	100.0%	64.4%	64.4%	64.4%	29.0%	37.0%	37.0%	3.6%	11.1%	100.0%	100.0%
	Mortality	100.0%	100.0%	81.2%	81.2%	81.2%	72.2%	75.0%	75.0%	67.1%	68.2%	100.0%	100.0%
mRNA	Infection	100.0%	100.0%	94.4%	94.4%	94.4%	90.9%	94.1%	94.1%	83.0%	86.2%	9.5%	43.5%
	Onward transmission	100.0%	100.0%	100.0%	100.0%	100.0%	100.0%	100.0%	100.0%	78.0%	100.0%	100.0%	100.0%
	Symptomatic desease	100.0%	100.0%	88.5%	88.5%	88.5%	80.4%	87.9%	87.9%	56.6%	66.0%	3.4%	6.7%

	Hospitalization	100.0%	100.0%	64.4%	64.4%	64.4%	29.0%	37.0%	37.0%	3.6%	11.1%	100.0%	100.0%
	Mortality	100.0%	100.0%	17.3%	17.3%	17.3%	15.4%	16.0%	16.0%	14.3%	14.5%	100.0%	100.0%

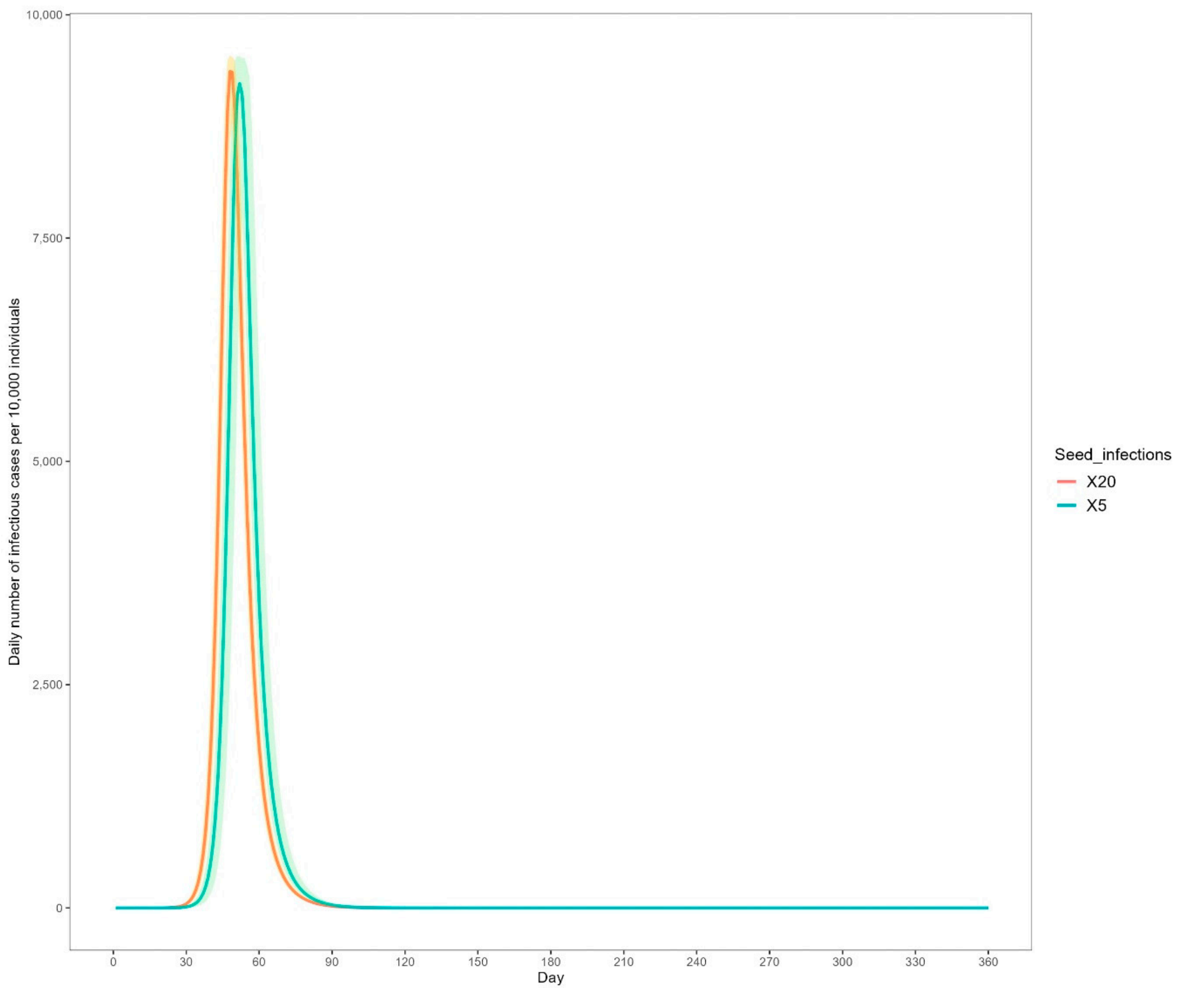

Figure S1. Projected SARS-CoV-2 Omicron burden in infections in China under scenarios with more initial introduced infections compared with baseline (20 vs 5)

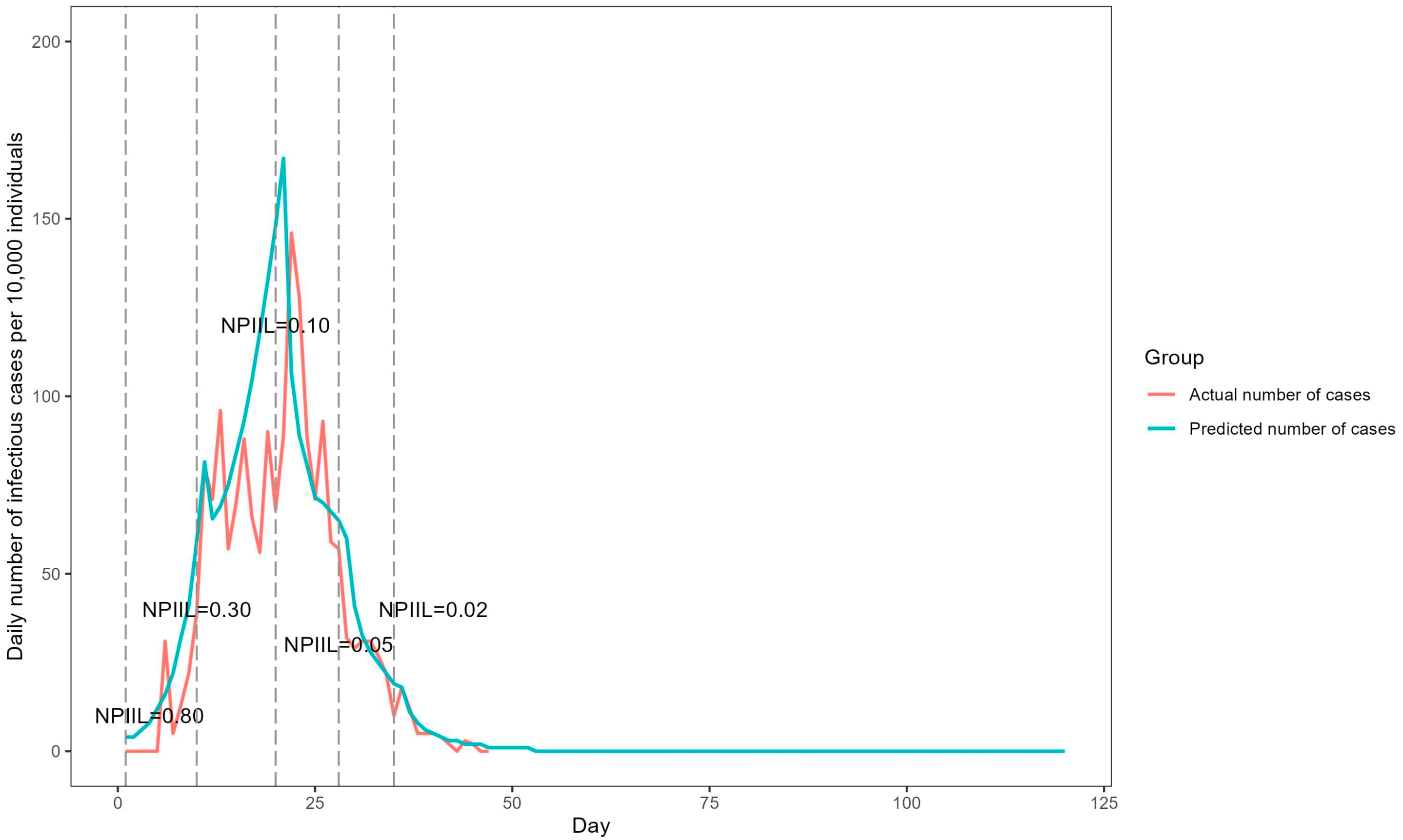

Figure S2. Daily infectious cases of SARS-CoV-2 Omicron variant in Macau

Note:

NPIIL: nonpharmaceutical intervention intensity level

NPIIL are between 0-1. NPIII equal to 0.01 means the strictest NPI is carried out, and vice versa

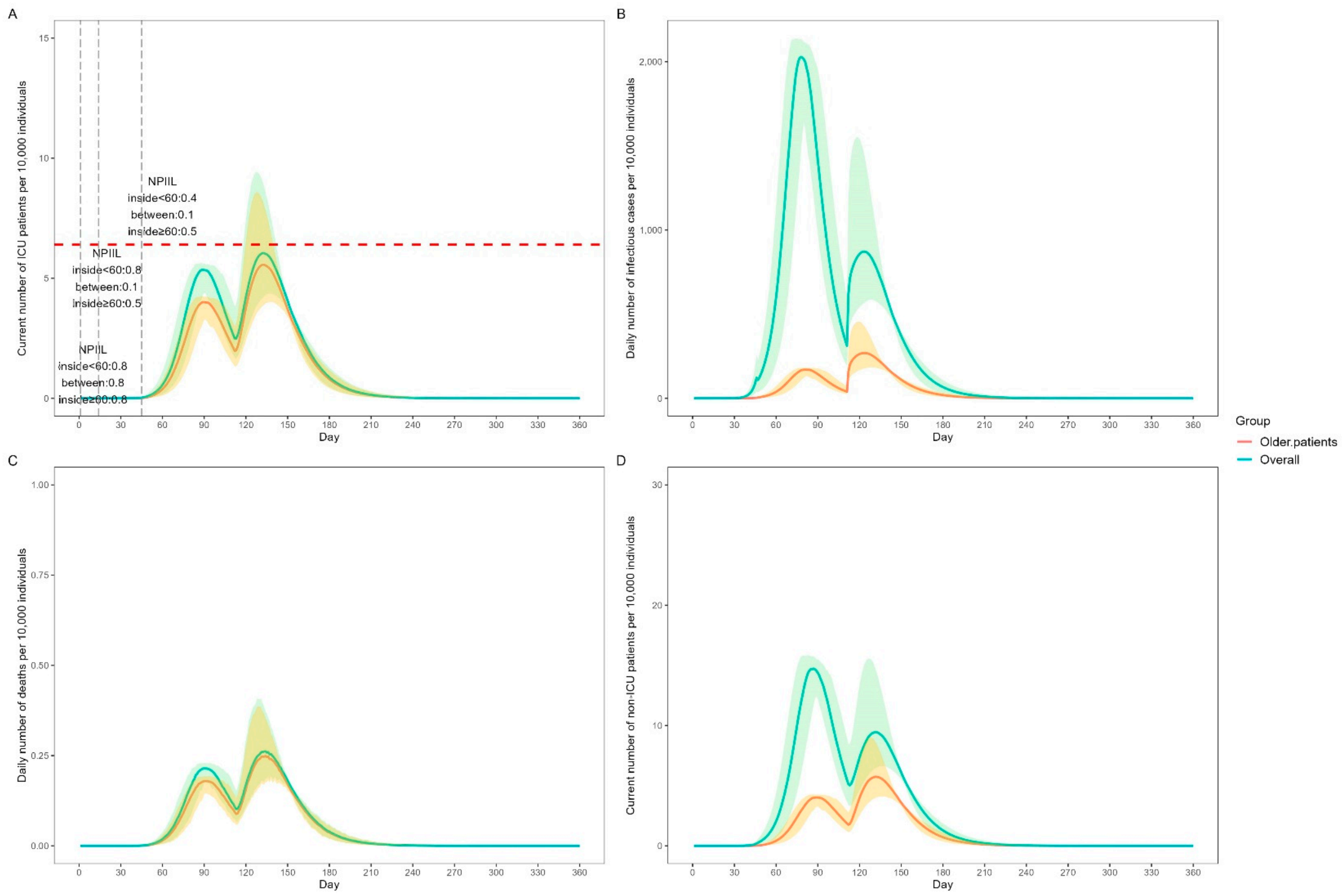

Figure S3. Prediction SARS-CoV-2 Omicron burden using different NPIIL of NIPs mitigation strategies at specific times among different age groups based on the positive scenario.
